# Nasal Cytology: A Easy Diagnostic Tool in Precision Medicine for Inflammation in Epithelial Barrier Damage in the Nose. A Perspective Mini Review

**DOI:** 10.3389/falgy.2022.768408

**Published:** 2022-04-06

**Authors:** Cristiano Caruso, Rossana Giancaspro, Giuseppe Guida, Alberto Macchi, Massimo Landi, Enrico Heffler, Matteo Gelardi

**Affiliations:** ^1^Unit of Internal Medicine and Gastroenterology, Department of Medical and Surgical Sciences, Agostino Gemelli University Polyclinic (IRCCS), Rome, Italy; ^2^Allergy Unit, Fondazione Policlinico A. Gemelli IRCCS, Rome, Italy; ^3^Unit of Otolaryngology, Department of Clinical and Experimental Medicine, University of Foggia, Foggia, Italy; ^4^Allergy and Pneumology Unit, A.O. S.Croce e Carle, Cuneo, Italy; ^5^Italian Academy of Rhinology Asst Settelaghi-University of Insubriae, Varese, Italy; ^6^Paediatric National Healthcare System, Torino, Italy; ^7^Personalized Medicine Center: Asthma and Allergology, Humanitas Research Hospital, Rozzano (MI), Italy; ^8^Department of Biomedical Sciences, Humanitas University, Pieve Emanuele (MI), Italy

**Keywords:** nasal cytology, biomarker, precision medicine, NARES, non-allergic rhinitis, chronic rhinosinusitis

## Abstract

Nasal cytology is a diagnostic tool that can be used in precision rhinology medicine. Particularly in non-allergic rhinitis and chronic rhinosinusitis forms it can be useful to evaluate biomarkers of both surgical or biological therapy and especially in the follow-up it must be used to predict the prognostic index of recurrence of nasal polyposis. All inflammatory cytokines are also linked to the presence of cells such as eosinophils and mastcells and nasal cytology is a non-invasive and repeatable method to assess the situation in real life.

## Introduction

Nasal cytology (NC) is a non-invasive, cheap and easy-to-apply diagnostic tool, which, in the last decade, has become an integral part of the diagnostic process of sinonasal disorders and their follow-up (1). Thanks to NC, several rhinopaties once defined as non-specific, have acquired nosological dignity and have been defined according to their cellular characteristics. Moreover, due to its reproducibility, NC allows to monitor the effectiveness of therapeutic strategies over time. This has significant therapeutic and prognostic implications, since the understanding of the cellular characteristics of diseases allows, in the era of Precision Medicine, not only to guarantee the patient tailored treatments, but also to establish patients' prognosis, avoiding false expectations of recovery and identifying *a priori* the most difficult to treat and more prone to relapse forms (2). As a matter of fact, according to NC findings and comorbidities, a Cytological-Clinical Grading (CCG) has been proposed for defining the severity of Chronic Rhinosinusitis with nasal polyps (CRSwNP) and the strictly correlated Prognostic Index of Relapse (PIR), in order to calibrate the therapeutic strategies to the probability of recurrence (3). Similarly, in the context of non-allergic rhinitis (NAR), although a specific degree has not yet been proposed to evaluate the refractoriness to treatments and the probability of relapse, the identification of inflammatory cells infiltrating nasal mucosa allows to predict patients' prognosis and to guarantee them suitable therapies.

### Nasal Cytology Procedure

NC technique consists in sampling, processing and microscope reading. In particular, cytological samples are obtained under anterior rhinoscopy, with the aid of an appropriate light source, by Nasal Scraping® (EP Medica, Italy), a sterile single-use curette. Samples are taken from the middle part of the inferior turbinate, where the optimal ratio of goblet cells to ciliated cells is 1:4, and immediately smeared on a glass side. After air-drying, samples are stained with May-Grunwald-Giemsa (MGG) and then read at optical microscopy, with a 1000x objective with oil immersion. A minimum of fifty fields is considered necessary to identify a sufficient number of cells. Accoding to the predominant type of inflammatory cell, four cytologic phenotypes are identified: neutrophilic, eosinophilic, mast-cell and mixed cellularity (eosinophil and mast-cells) (4). This painless procedure is repeatable and inexpensive, so it can be performed on the same patient periodically to monitor the progress of the inflammatory infiltrate.

### Epithelial Barrier Damage

The nasal mucosa has traditionally been considered a mere physical barrier between the host and its environment. Nowadays, it is becoming increasingly clear that nasal mucosa is metabolically active and plays a crucial role in maintaining the immunological barrier, producing various inhibitory substances and secretory IgA (5). While histological samples allow to directly detect any defects in the integrity of the nasal mucosal barrier, nasal cytology samples allow to indirectly detect such alterations. In particular, the Hyperchromatic Supranuclear Stria (SNS) is considered a specific cytological marker for the anatomic and functional integrity of ciliated cells and, therefore, for the mucosal barrier integrity. Indeed, since diseases affecting nasal mucosa epithelium determine its rearrangement, ciliated cells manifest distress phenomena including the disappearance of the hyperchromatic SNS. Therefore, the absence of the SNS is considered a useful prognostic sign of nasal disorders (6). Furthermore, among the mechanisms responsible for epithelial toxicity during allergic inflammation, the predominantly eosinophilic inflammatory infiltrate has been shown to be responsible for the barrier damage. As a matter of fact, eosinophils secrete several cationic proteins, including major basic protein (MBP), which induces a marked decrease in the number of desmosomes and an exfoliation of epithelial cells (7). The noteworthy role that eosinophils play in the ephitelial barrier damage has been clearly described in asthmatic patients, in which a strong correlation has been found between the eosinophilic infiltrate, MBP and the presence of “Creola bodies,” defined as clusters of apoptotic epithelial cells resulting from MPB-induced exfoliation (7). In AR, the increased permeability of the epithelium, due to damage of the cell junctions, as well of the exfoliation of the epithelial cells allow allergens and other noxious substances to penetrate the barrier and cause the main symptoms of sino-nasal disease (8). Indeed, the direct contact of the latter substances with the trigeminal irritant receptors causes the nasal hyperactivity that characterizes vasomotor rhinitis (9).

### The Role of Nasal Cytology in the Diagnosis of NARs

NC has contributed substantially to the understanding of the different forms of rhinitis, traditionally classified, based on the etiology, in infectious, inflammatory, vasomotor, medicamentous, hormonal, occupational and atrophic. In particular, NC has allowed to give a precise identity to NARs, once defined as “non-specific” due to the capacity of nasal mucosa to respond to non-specific stimuli, such as temperature, humidity and odors, unlike allergic rhinitis (AR), which is induced by type 1 hypersensitivity response following the exposure to allergens in sensitized individuals (10, 11). Therefore, NC allowed differentiating the pathologies belonging to this heterogeneous group of disorders, accumulated by similar symptoms, including rhinorrhea, sneezing, itching and nasal obstruction, variously associated, but characterized by different inflammatory cell infiltrate.

**Figure 1:**
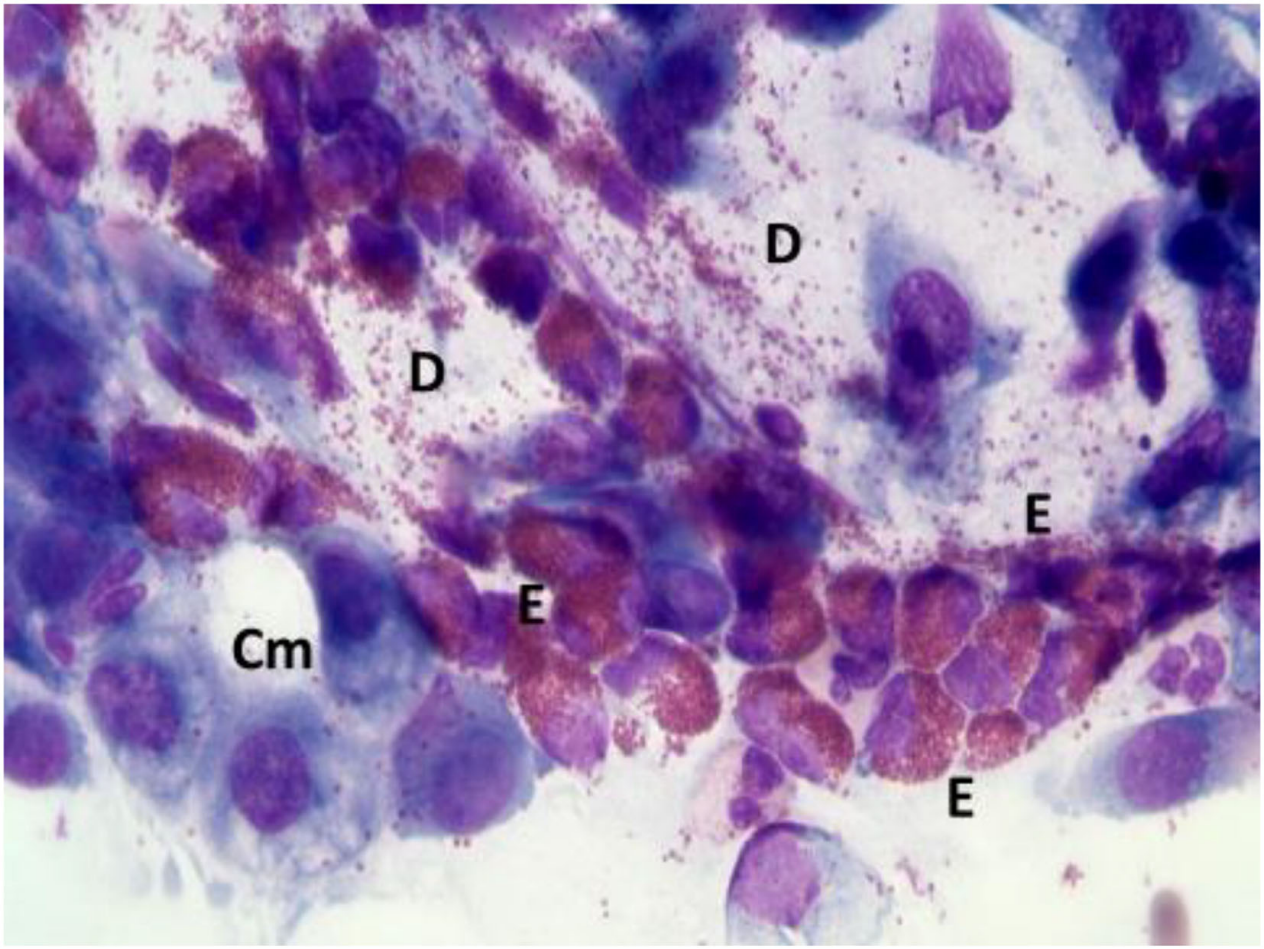
Nasal cytology of NARES. E, eosinophil; D, degranulation; Cm, calicifom mucous cells. MGG staining. Magnification 1000x.

**Figure 2:**
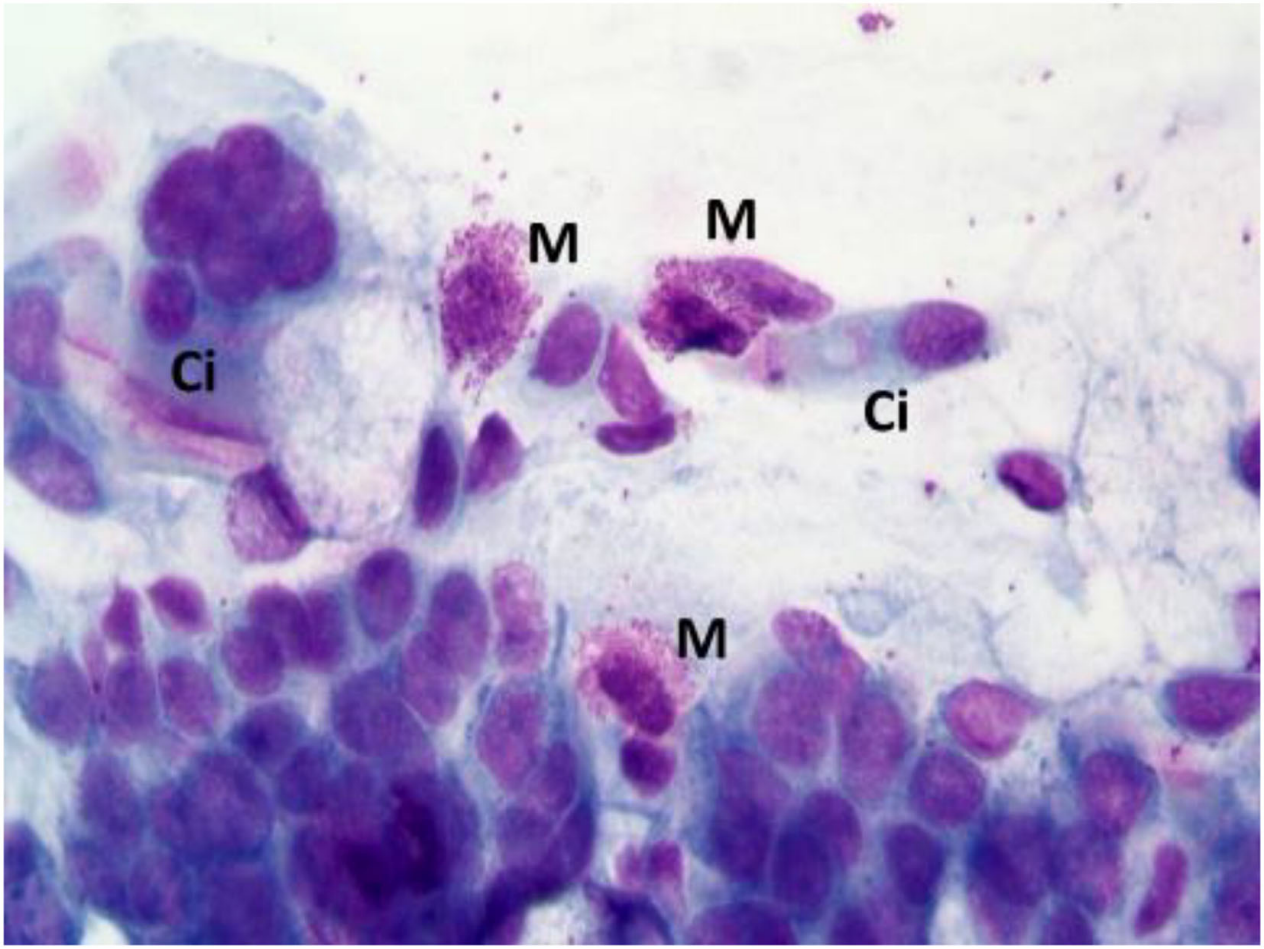
Nasal cytology of NARMA. M, mast cell; D, Degranulation; Ci, ciliated cell. MGG staining. Magnification 1000x.

**Figure 3:**
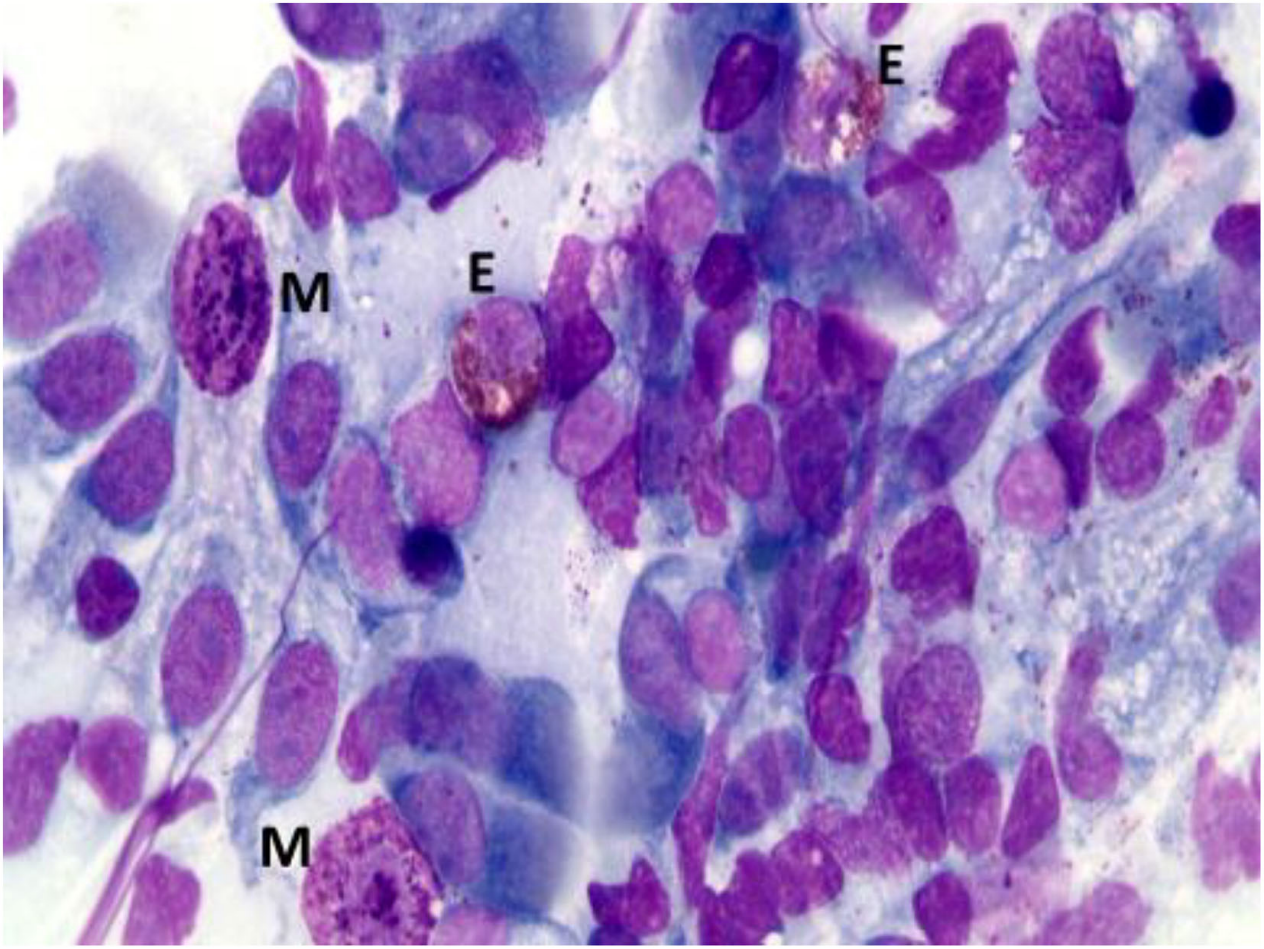
Nasal cytology of NARESMA. E, eosinophil; M, mast cell; D, degranulation. MGG staining. Magnification 1000x.

According to NC findings and to the predominant infiltrating cell types, NAR is nowadays classified into NARES (NAR with eosinophils) (Figure 1), NARMA (NAR with mast cells) (Figure 2), NARNE (NAR with neutrophils) and NARESMA (NAR with eosinophils and mast cells) (Figure 3). The different types of NARs are characterized by different prognosis and responsiveness to medical treatments. Specifically, NARESMA is the most difficult to treat form, frequently associated with the development of CRSwNP (12).

### Allergic Rhinitis and Overlapping Rhinitis

The allergic reaction consists of a so-called early phase, mainly mediated by histamine, and a late phase, caused by inflammatory cells. From a cytological point of view, these responses are characterized by the presence of inflammatory cells infiltrating the nasal mucosa, including eosinophils, mast cells, neutrophils and plasma cells, which release several chemical mediators, responsible for the main symptoms of AR (itching, nasal congestion, runny nose, sneezing, etc.) (13).

**Figure 4:**
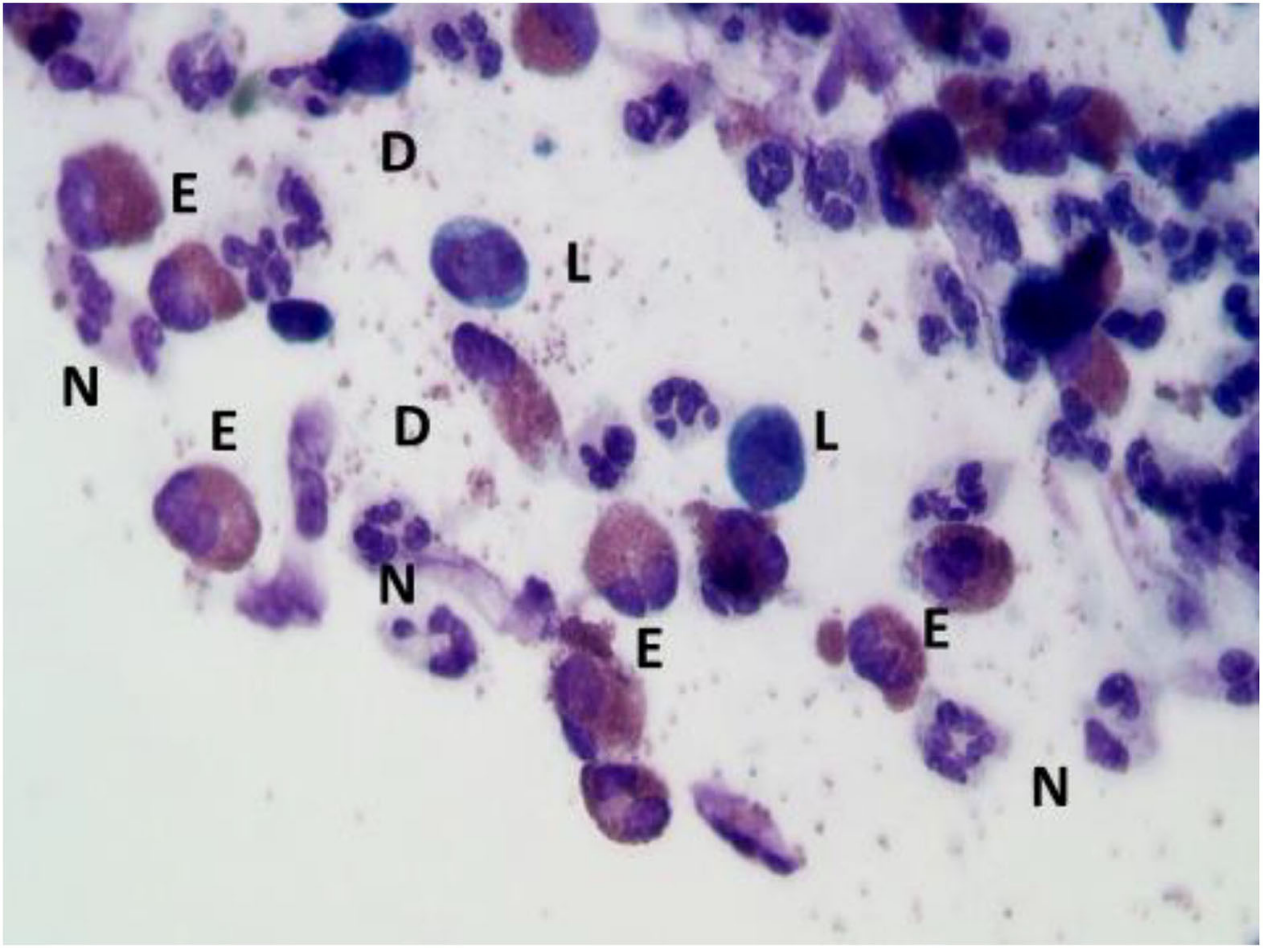
Nasal cytology of AR. E, eosinophil; N, neutrophils; L, lymphocytes; D, degranulation. MGG staining. Magnification 1000x.

In the context of allergic rhinitis, NC findings vary according to the type of allergens to which patients' are sensitive. In particular, in patients with perennial AR, characterized by an allergen exposure of low intensity but persistent in time, rhinocytograms show “minimal persistent inflammation,” represented by a persistent infiltration of neutrophils and only minimally by eosinophils. On the contrary, in seasonal AR rhinocytograms change depending on whether patients are examined during the exposure of allergens or not. During the pollen season, patients present all the clinical signs of AR and nasal cytology show neutrophils, lymphocytes, eosinophils and MCs, largely degranulated (Figure 4); conversely, out of season of exposure, the patient clearly present a clinical and cytological “silence”.

In addition, nasal cytology has brought to light an important problem: since AR and NAR are two separate entities, they can coexist in the same patient in up to 15–20% of cases. Overlapping rhinitis (ORs) are considered traps for allergists and otolaryngologists, since they are often misdiagnosed, resulting in the failure of medical and surgical strategies (14). In fact, patients with AR tend to be treated as purely allergic and, eventually, with Sublingual Immunotherapy (15). Since this therapeutic strategy targets specifically allergic processes, patient with overlapped rhinitis experiences only a partial benefit, despite expectations.

This further emphasizes the importance of identifying, with the aid of the NC, the specific pathology of which patients are affected, since only a correct diagnosis can guarantee an effective therapy.

### Nasal Cytology in CRSwNP Patients' Work-Up

Chronic rhinosinusitis (CRS), affecting approximately 5 to 12% of the general population, is a chronic inflammatory disease of the nasal mucosa, characterized by the presence of cardinal symptoms, such as nasal obstruction, rhinorrhea, facial pain and hyposmia or anosmia lasting longer than 12 weeks. According to EPOS 2020 Guidelines, the diagnosis of CRS also requires the presence of both endoscopic and radiological signs, including the presence of nasal polyps, mucopurulent discharge from the middle meatus and/or mucosal edema (16). Given the significant burden on society, due to the impaired quality of life (QoL), losses in productivity and high direct and indirect health care costs, the understanding of pathophysiological processes underlying CRS and, therefore, of effective therapeutic strategies is of utmost importance (17). According to the presence or the absence of nasal polyps, CRS has traditionally been classified in two major phenotypes: CRS with nasal polyps (CRSwNP) and CRS without nasal polyps (CRSsNP). In particular, among CRS patients, 31% of them suffer from CRSwNP, which tends to be a clinically more severe disease, compared with CRSsNP. CRSwNP results from the dysfunctional interplay between host immunity, defective epithelial barrier, and environmental factors (18). In order to better characterize the disease and guarantee tailored and effective treatments, the identification of the different endotypes is essential. As a matter of fact, over the past 20 years, advances in the field of technology and scientific research have dramatically changed the clinical approach to diagnosis and treatment of rhinitis, paying greater attention to inflammatory infiltrate and to the cytokine inflammatory patterns that establish CRS. In this perspective, several diagnostic procedure have been introduced in order to refine the diagnostic accuracy, including fiberoptic endoscopy, immunohistochemistry, biomarkers of inflammation and NC (19, 20). Among these procedures, nasal cytology (NC) has been widely recognized as a non-invasive, cheap and easy-to-perform diagnostic tool, which allows to define predominant cells of the nasal inflammatory infiltrate and, thus, the endotype of CRS, with significant diagnostic and prognostic implications. In fact, NC allows to detect and quantify the predominant cell population within the nasal mucosa at a given time, with the primary objective of making a correct differential diagnosis and monitoring the effects of treatments over time.

Moreover, in order to further practice Precision Medicine, among the potential applications of nasal cytology there would the possibility of evaluating the presence of several biomarkers, defined as indices of severity or predictors of recurrence of a disease, in cytological samples. Typically, biomarkers are detected by histological investigations, which are more invasive and expensive than nasal cytology. Nevertheless, the identification of the same biomarkers would also be possible on cytological samples, carrying out, for example, immunofluorescence and confocal laser microscopy.

#### Cytological-Clinical Grading

CCG assesses the severity of CRSwNP as a function of NC findings and comorbidities. In particular, each comorbidity corresponds to a certain score: ASA sensitivity corresponds to 1, asthma to 2, allergy to 3, ASA sensitivity combined to asthma to 3. Likewise, a score is assigned to each inflammatory cell pattern and, in particular neutrophilic infiltrate corresponds to 1, mast cell to 2, eosinophilic to 3, mixed eosinophilic-mast cell to 4. The final CCG score corresponds to the sum of the scores attributed to the endotype, represented by the predominant cellular infiltrate, and to the phenotype, represented by the various comorbidities. The severity of CRSwNP is strictly associated with the refractoriness of the disease. In fact, the CCG corresponds to the Prognostic Index of Relapse (PIR), which is precisely the prognostic expression of CCG and in particular, as the score increases, from one to ten, the probability of relapse and non-responsiveness to medical and surgical treatments increases. Therefore, a patient with multiple comorbidities and a predominantly eosinophilic or mixed eosinophilic-mast cell infiltrate is more prone to relapse. As a matter of fact, the degree of eosinophilic infiltration is widely recognized as one of the main indicators of severity and predictors of relapse and several biomarkers of eosinophilic inflammation have been identified (21). NC has shown that not only eosinophils but also mast cells are involved in contributing to the severity of the disease, orchestrating the inflammation underlying CRSwNP and producing a series of type 2 inflammatory cytokines (22). This cytologic point of view is of utmost importance, since in the era of Precision Medicine, several biological agents have been approved or are under evaluation for the treatment of CRSwNP (23, 24). These agents exhibit a broad mechanism of action on type 2 inflammation, acting on several targets. Hence the importance of clearly defining the CRSwNP endotype with diagnostic tools available today, first of all NC, and choosing a suitable treatment.

In this context, NC represents a useful tool for the follow-up of CRSwNP since it allows to monitor the changes of the nasal inflammatory infiltrate over time and to evaluate the effectiveness of the chosen treatment.

#### The Role of NC in Detecting Biofilm

Among the factors implicated in the pathogenesis of CRSwNP, *Staphylococcus aureus* has been shown to play a crucial role, impairing the function of the epithelial barrier, producing enterotoxins and serine protease-like protein and enhancing the production of Th2 cytokines through rapid induction of of epithelial-derived innate cytokines. The latter promote Th2 responses through the development of innate lymphoid cells and the induction of mast cells (25).

Staphylococcal colonization is responsible for bacterial dysbiosis and leads to biofilm formation and recalcitrant disease (26). Biofilms consist of microbial communities embedded in an extracellular matrix, composed of a complex series of extracellular polymeric substances (EPS) (27). This matrix confers evolutionary advantages for the pathogen microorganisms, such as extreme high resistance against chemical agents and host immune reactions, due to the physical barrier formed by the matrix that blocks the diffusion of antibiotics, superoxides, immuno-globulins, and complement components. Biofilm-positive forms of CRSwNP are characterized by a predominant pattern of neutrophil granulocytes, mononuclear and dendritic cells. These features might explain surgical failures and high recurrence rates in CRSwNP (28).

**Figure 5:**
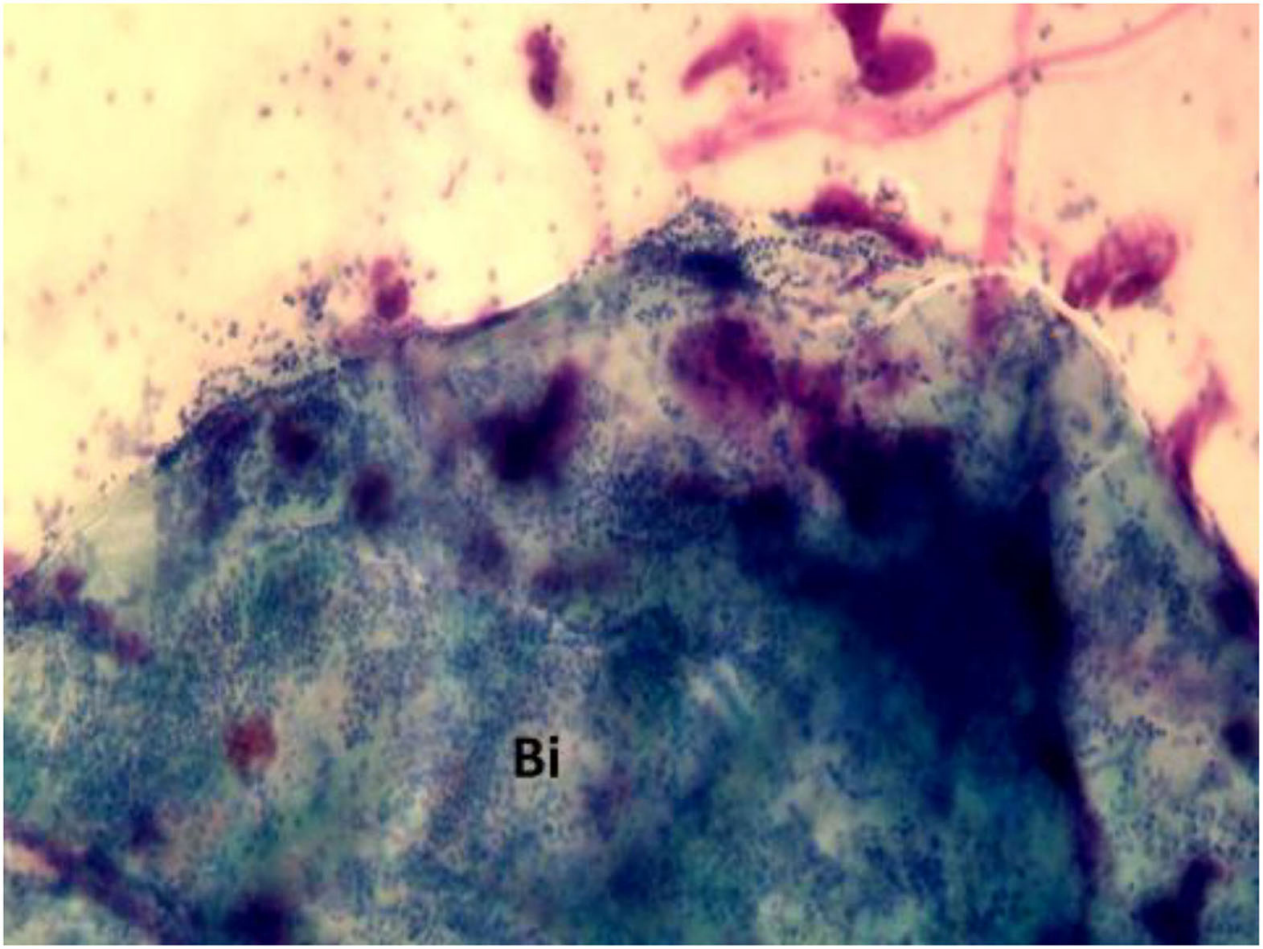
Nasal cytology in a patient with CRSwNP, showing “cyan” spots. Bi, biofilm. MGG staining. Magnification 1000x.

Biofilm can be detected by highly sensitive techniques including scanning electron microscopy (SEM), transmission electron microscopy (TEM), confocal laser scanning microscopy (CLSM) combined with live/dead staining or fluorescent *in situ* hybridization (FISH) (29). However, beyond these sensitive but very expensive techniques, NC represents an inexpensive procedure that allows for direct detection of the biofilm. In particular, in rhinocytograms, biofilms appear as spots of “cyan,” called “infectious spots,” which include bacterial colonies and/or fungal spores, positive for staining to periodic acid Schiff, confirming the polysaccharide component of these formations (30) (Figure 5).

Biofilm detection is a crucial step in CRSwNP patients' work-up, since it represents a target in treatment of chronic rhinosinusitis. In this context, amoxicillin-clavulanic acid and levofloxacin have been shown to be potent antibiofilm agents in CRSwNP patients (31).

## Conclusion

The rhino-allergological patient needs a precise diagnostic framework as well as to be closely monitored over time. In the era of Precision Medicine, there is a growing awareness that only a correct identification of the cellular and inflammatory pathways that underlie a specific disease can guarantee the patient the appropriate therapeutic strategy (32). The choice of the most suitable treatment, tailored to the patient, among all those available today represents a constant and of primary importance challenge. In this context, among all the diagnostic tools currently available to the otolaryngologist, NC represents a non-invasive, economical and reproducible procedure, which allows to precisely frame the patient and therefore to correctly choose the therapeutic strategy. In addition, by identifying the most severe and prone to relapse forms, NC findings allow to avoid false expectations of complete recovery and to improve patients' compliance. Eventually, NC constitutes even a valid aid for rhinological follow-up, since it allows to monitor the nasal inflammatory infiltrate and to evaluate its changes following medical and surgical treatments.

## Author Contributions

CC and GG conceived the work. RG and MG processed the editing and images. AM, ML, and EH have collaborated on the references. All authors contributed to the article and approved the submitted version.

## Conflict of Interest

The authors declare that the research was conducted in the absence of any commercial or financial relationships that could be construed as a potential conflict of interest.

## Publisher's Note

All claims expressed in this article are solely those of the authors and do not necessarily represent those of their affiliated organizations, or those of the publisher, the editors and the reviewers. Any product that may be evaluated in this article, or claim that may be made by its manufacturer, is not guaranteed or endorsed by the publisher.
